# Does shockwave therapy lead to better pain and function than sham over 12 weeks in people with insertional Achilles tendinopathy? A randomised controlled trial

**DOI:** 10.1177/02692155241295683

**Published:** 2024-12-20

**Authors:** Baraa Alsulaimani, Luke Perraton, Patrick Vallance, Tim Powers, Peter Malliaras

**Affiliations:** 1 Department of Physiotherapy, School of Primary Health Care, 2541 Monash University, Frankston, Victoria, Australia; 2 Faculty of Education, 2541 Monash Data Futures Institute, Melbourne, Victoria, Australia

**Keywords:** Insertional Achilles tendinopathy, extracorporeal shockwave therapy, tendinopathy

## Abstract

**Objectives:**

To investigate the efficacy of adding radial extracorporeal shockwave therapy or sham to exercise for people with insertional Achilles tendinopathy.

**Design:**

A two-armed, parallel-group, explanatory, single-centre, randomised controlled trial within a superiority framework.

**Setting:**

Private clinic.

**Participants:**

People diagnosed with insertional Achilles tendinopathy who were over 18 years old with a symptom duration of greater than 3 months.

**Intervention:**

A total of 76 people were randomly assigned (one-to-one ratio) to receive three sessions of radial extracorporeal shockwave therapy or sham to the affected side (or most affected side if bilateral). All participants received identical education and exercise.

**Outcome measures:**

The primary outcome was the Victorian Institute of Sports Assessment – Achilles questionnaire. Measures were recorded at baseline, 6 weeks and 12 weeks.

**Results:**

At 12 weeks, the questionnaire data were available for 37 people (96%) in the radial extracorporeal shockwave therapy group and 36 people (95%) in the sham group. For the primary outcome, we found no evidence for between-group differences at 6 (3, 95% confidence interval −4.6–10.5) or 12 weeks (4.6, 95% confidence interval −2.5–11.6). There was also no evidence for a between-group difference for any secondary outcome measures at either 6 or 12 weeks (*p* > .05). No serious adverse events were reported.

**Conclusion:**

The addition of radial extracorporeal shockwave therapy to exercise and education did not lead to improvements in pain, function or other outcomes compared to sham at 6 or 12 weeks among people with insertional Achilles tendinopathy.

**ANZCTR Reg No:** ACTRN12620000035921

## Introduction

Achilles tendinopathy is a common overuse condition characterized by activity-related pain, disability, swelling and local tissue pathology.^1^ Achilles tendinopathy can severely reduce physical capacity, including walking and running^1^ and accounts for up to 9% of all running injuries.^2^ There are two related variants – midportion and insertional Achilles tendinopathy. Insertional Achilles tendinopathy has been reported to be challenging to manage compared to midportion Achilles tendinopathy.^1^ This may be because of poor blood supply to the tendon insertion^3^ and compression of the tendon against the bone that occurs at this site.^1^

Although exercise is the recommended first-line treatment for Achilles tendinopathy^4^ up to 44% (in a sedentary population) and 75% (using undefined exercise) of people may not respond to this intervention.^1^ A common adjunct therapy for insertional Achilles tendinopathy is radial extracorporeal shockwave therapy (rESWT). Radial extracorporeal shockwave therapy involves high-frequency pressure waves delivered to the tendon.^5^ Despite being commonly used clinically,^1^ evidence for radial extracorporeal shockwave therapy among people with insertional Achilles tendinopathy is not certain. Only one adequately powered study has investigated whether radial extracorporeal shockwave therapy is superior to sham when added to exercise. Mansur et al.^6^ utilised an eccentric training program that involves 90 repetitions (45 with the knee bent and 45 with the knee straight). There were no between-group differences for the primary composite pain and function outcome at any time point up to 24 weeks. However, the absence of findings may be explained by all participants being asked to stop running and sports activities for 8 weeks regardless of pain levels, which is not currently recommended by the clinical practice guidelines.^1,4^ Considering the widespread use of radial extracorporeal shockwave therapy in clinical practice and the importance of education, it is important to re-evaluate its efficacy compared to placebo in the current context of recommended care.

The primary aim of this trial was to investigate the efficacy of radial extracorporeal shockwave therapy or sham in combination with recommended exercise and education on pain and function outcomes at 6 and 12 weeks (primary endpoint) among people with insertional Achilles tendinopathy. The secondary aim was to investigate the efficacy of the radial extracorporeal shockwave therapy or sham at the same timepoints on overall pain, physical activity, psychological (kinesiophobia, catastrophising, pain self-efficacy) and health-related quality of life outcomes.

## Methods

This was a two-armed, parallel-group, explanatory, single-centre, randomised controlled trial. All treatments were delivered in a private clinical setting in Melbourne, Australia. The trial used a superiority framework to determine whether radial extracorporeal shockwave therapy is superior to sham in relation to pain and function outcomes at 6 and 12 weeks. The trial protocol was prospectively registered (ACTRN12620000035921) and the statistical analysis plan was published prior to undertaking statistical analysis (https://osf.io/sj8q4/). The trial was reported in accordance with the Consolidated Standards of Reporting Trials statement^7^ and tendinopathy reporting standards.^8^ The template for treatment description and replication was used to guide reporting of the radial extracorporeal shockwave therapy treatment as well as the exercise and education provided to both groups.^9^ Ethics approval was granted by the Monash University Human Ethics Committee (Project ID: 21015).

The trial was powered to detect a minimal clinically important difference of 6.5 points on the Victorian Institute of Sports Assessment – Achilles questionnaire.^10^ The assumed standard deviation for the Victorian Institute of Sports Assessment ­– Achilles questionnaire was 14 based on the largest observed standard deviation in the meta-analysis of radial extracorporeal shockwave therapy for Achilles tendinopathy.^11^ Based on the minimal clinically important difference and standard deviation, Cohen's *d* was converted to an *f*^2^ effect of 0.13.^12^ G-Power 3.6^12^ was used to estimate the sample size required for when specifying three tested predictors and controlling for three covariates. This sample size was then adjusted to account for the repeated timepoints used in the linear mixed model (adjusting for intercluster correlation and average cluster size).^12^ The final sample size required was 74 participants (accounting for 10% attrition) with a *p* value of 0.05 and 80% power.

Participants aged 18 years or older with unilateral or bilateral insertional Achilles tendinopathy for more than 3 months were recruited via local clinicians (General practitioners, sports physicians, allied health clinicians) and paid social media advertising. To be diagnosed with insertional Achilles tendinopathy, participants met the following criteria: (a) pain localised to the posterior calcaneum; (b) progressive commencement of pain in this area; (c) pain within or following Achilles tendon loading activities (e.g., walking, running); and (d) absence of signs or symptoms of posterior ankle impingement or ankle joint pathologies (e.g., osteoarthritis). Ultrasound imaging was used to confirm the presence of Achilles tendon insertional pathology which could include hypoechoic areas either with or without tendon thickening or fluid inside the retrocalcaneal bursa. Participants were excluded if they had previously received radial extracorporeal shockwave therapy for any condition or had a history of Achilles tendon surgery or rupture (on the most painful side if pain was bilateral), inflammatory arthropathy (e.g. ankylosing spondylitis), neurological disorders that may affect calf function (e.g. Parkinson's disease, stroke), genetic connective tissue disorders (i.e. Ehlers–Danlos syndrome, Marfan's syndrome), fluoroquinolone antibiotics use over the previous 2 years, or a serious mental health condition or other medical/social reasons (e.g. homelessness) that could impact on safety or adherence with the treatment.

Participants were recruited (from April 2021 to November 2022) via the following channels: (a) a social media that included Facebook and Instagram posts; and (b) advertisement of the trial to the researchers’ large network of clinical partners.

Participants were block-randomised (computer-generated permuted blocks of 4, 6 or 8) into radial extracorporeal shockwave therapy or sham at their baseline visit. Randomisation was done by a researcher (remote to the clinic centre) who had no contact with the participants. This researcher informed the physiotherapists delivering the treatments (via text message) of the group allocation. The physiotherapists delivering the radial extracorporeal shockwave therapy or sham were not masked, but they followed a script (described below) to minimise differences in the delivery of each treatment. A separate physiotherapist who was masked to group allocation delivered exercise and education to both groups and administered the outcome measures. Participants were masked for group allocation.

Participants were scheduled to receive three radial extracorporeal shockwave therapy or sham treatments (5–10 days apart). During the treatment, participants were lying prone. Treatment focused on the most painful part of the Achilles tendon insertion according to the patient while the investigator palpated the calcaneum (the region was between 3 and 4 cm^2^ and marked with a hypoallergenic marker). Three physiotherapists with adequate training and all with more than a year's experience using radial extracorporeal shockwave therapy delivered both treatments.

To maintain participant masking, the following steps were taken: (1) use of identical radial extracorporeal shockwave therapy procedures for both groups, including length of application, pressure of probe applied to tendon surface, positioning of participant and use of gel; (2) the sham extracorporeal shockwave therapy device was identical to the radial extracorporeal shockwave therapy device (including noise), with only the removal of the device minimising pressure. We also staggered interventions so that participants could not interact within the treatment facility and share experiences about treatments received. Participants received 3000 shocks of radial extracorporeal shockwave therapy or sham at a frequency of 10 hertz with a commercially available device (Intellect RPW2, Chattanooga, USA). The starting point for pressure was 2 bars for all participants. To conduct pressure waves to the tendon aqueous gel was used. The minimum target self-reported pain was ≥ 5 (numeric rating scale: 0 = no pain, 10 = worst pain imaginable). If self-reported pain was <5 on this scale, the radial extracorporeal shockwave therapy probe was changed to a different area within the area of interest (as defined above). If the applicator was slowly moved throughout all parts of the region of interest and the pain remained <5, then the radial extracorporeal shockwave therapy pressure setting was raised (in increments of one bar [the maximum bar pressure on the device was 5]) to accomplish the target self-reported pain level.

This process (first exploring the region and interest and then increasing the bar pressure incrementally) was repeated as needed up until the shock dose of 3000 was done (the physiotherapists asked for a verbal report of pain experienced every 10 seconds during the 5-minute treatment period). The procedure for the sham treatment was identical but because the treatment was devoid of pressure waves and therefore far less painful, participants generally all reached the maximum bar pressure of 5.

Identical exercise and education were delivered to both groups by an appropriately trained physiotherapist and researcher. Education included information about Achilles tendinopathy types and symptoms, risk factors, causes of pain and evidence-based management.

**Figure 1.**
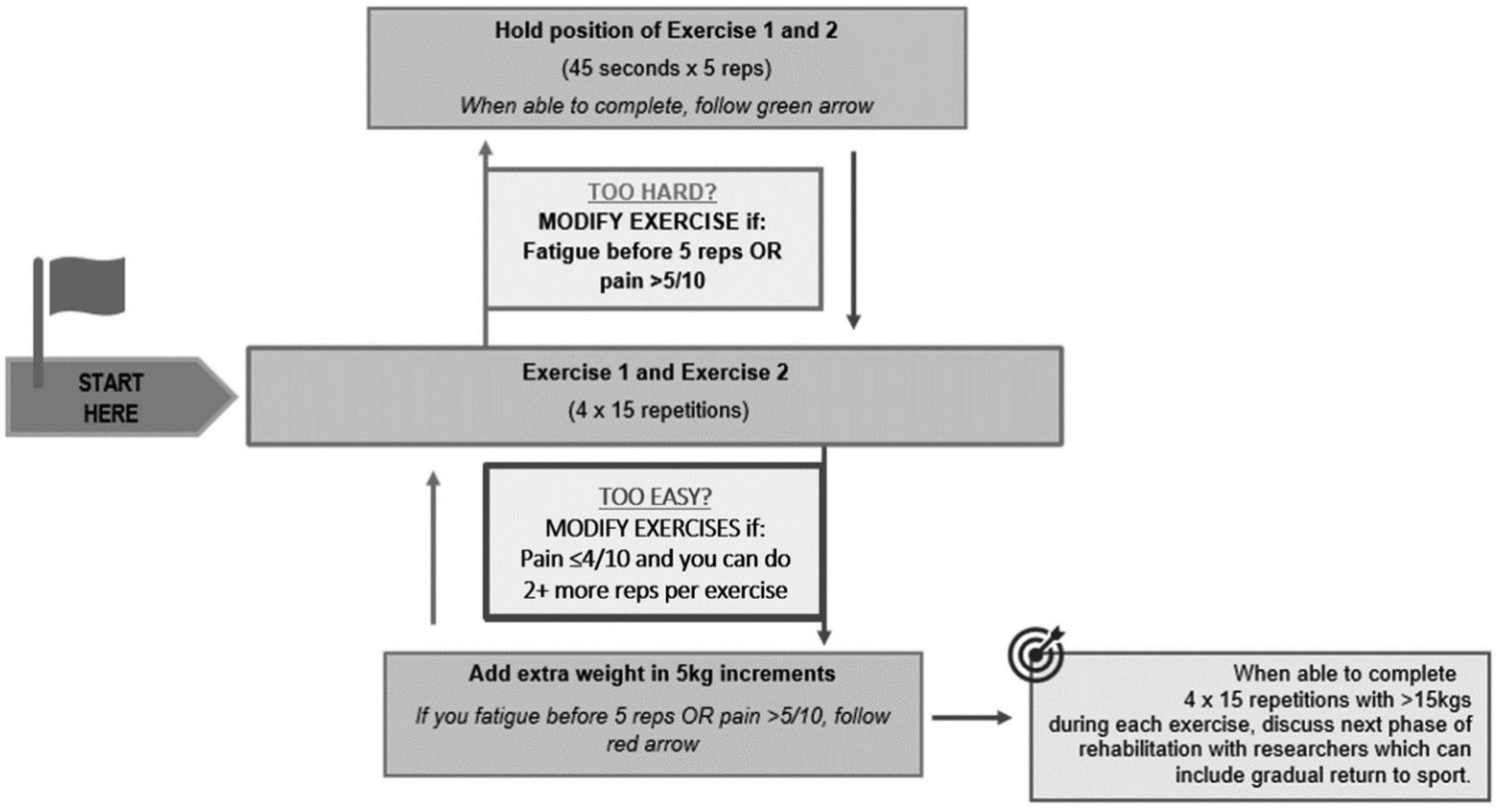
Description of straight and bent knee exercises.

The exercise treatment was based on empirical evidence^1,13^ and involved four sets of 15 repetitions of calf raises with knees bent (30° of knee flexion) and knees straight performed three times (every second day) a week on flat ground with a 2-minute rest between sets and 1 minute between exercises. The progression of exercise was tailored to individual goals (Figure 1). Participants started in a standing position on a flat surface next to a wall. They could use the wall for balance if needed. While standing on one leg, participants were instructed to lift the heel as high as possible on the weight-bearing leg, rising up onto the toes. They were then advised to slowly lower the heel down and ensure they kept the knee straight throughout the exercise. If pain was acceptable (≤4 out of 10) and they were not fatigued with the prescribed repetitions, they were instructed to add weight in 5-kg increments (using items like rice packets, books, and bricks in a backpack). They were also advised to continue increasing the weight by 5 kg up to the deemed maximum weight, depending on their activity goals identified in the first session (walking [15 kg], running or other sport [25 kg]).

Participants could continue with current walking, running and sports activities if pain during these activities was acceptable (≤4 out of 10). Otherwise, they were advised to reduce the volume of these activities by approximately 50%. When pain during these activities became acceptable, they were advised to gradually return to their goal volume of running or sport.

Pain up to a level of 4 out of 10 (numerical pain rating scale, 0 = no pain, 10 = worst pain imaginable) was acceptable during the exercise. If pain during exercise was acceptable and participants reported two or more repetitions in reserve at the end of the prescribed sets, then they were instructed to add weight in 5 kg. If pain was not tolerable (≥5), they were directed to proceed back to an isometric exercise. Pain increase after exercise was also acceptable if it returned to pre-exercise levels within 24 h (Figure 1). If the pain was beyond a 5 out of 10, participants were advised to stop and recommence the following day.

The exercise and education were delivered via printed or electronic pamphlets (Supplementary Files 1 and 2) and website-based videos demonstrating the exercises. During the first session, the physiotherapist spent 30­–40 minutes discussing all aspects of the education pamphlet with the participant (pausing to check comprehension and whether there were questions) and prescribing the exercises. When participants returned for their second and third radial extracorporeal shockwave therapy or sham treatments, the masked physiotherapists checked and progressed the participants’ exercise as required, checked their understanding of the education and answered any questions. There were similar follow-up sessions via teleconference with the same physiotherapist at weeks 6 and 12. Participants had the physiotherapists’ contact details and were advised to contact if they had any problems, such as an adverse event. They were advised to refrain from other treatments and non-steroidal anti-inflammatory medications but were advised to take paracetamol (up to 4 g/day) for pain relief if needed.

At baseline, demographic data (i.e. age, sex, weight, height, body mass index), healthcare history (i.e. medical history) and specific Achilles tendinopathy history (i.e. site, duration, previous treatment) were recorded. During radial extracorporeal shockwave therapy, the maximum bar pressure as well as the maximum self-reported pain level were recorded. The success of masking was assessed immediately after the initial radial extracorporeal shockwave therapy or sham session by asking participants which group they thought they were allocated to (radial extracorporeal shockwave therapy, sham, not sure).

Primary and secondary outcome measures were assessed at baseline, 6 and 12 weeks via email or text link to an online questionnaire. The 12-week point in time was chosen as the primary endpoint as some of the effects of radial extracorporeal shockwave therapy may occur over a number of weeks.^14,15^ The Victorian Institute of Sports Assessment – Achilles questionnaire is a 10-item, disease-specific tool assessing Achilles tendon pain, participation (in sport or physical activity) and disability (impaired sport and physical activity in relation to pain) domains (0 points = worst score; 100 points = best score).^16^ The minimal clinically important difference is 6.5 points on Victorian Institute of Sports Assessment – Achilles questionnaire.^10,17^ Secondary outcomes included: (1) the worst pain (100 mm visual analogue scale) experienced over the last 24 hours; (2) physical activity in the previous week evaluated with the 7-day Recall Physical Activity Questionnaire;^18^ (3) kinesiophobia assessed with the 11-item Tampa Scale of Kinesiophobia;^19^ (4) pain catastrophising assessed using the Pain Catastrophising Scale;^20^ (5) pain self-efficacy using the Pain Self-Efficacy Questionnnaire;^21^ and (6) health-related quality of life measured with the EuroQol-5D-5L (including assessment of the five domains of mobility, self-care, usual activities, pain/discomfort and anxiety/depression as well as overall health state from 0 [worst health state imaginable] to 100 [best imaginable health state] using a 100-mm visual analogue scale).^22,23^ We also assessed exercise adherence (number of sessions per week completed on average over the last 6 weeks), and other treatments (professionals seen, number of sessions, and other treatments over the last 6 weeks). Self-reported adverse events were assessed via a custom questionnaire at 6 and 12 weeks (asking about the type of event, how long it lasted, and what treatment was needed). These were reported as the number of people experiencing a serious adverse event [defined as results in death, life-threatening complication, hospitalisation, surgery, permanent or temporary physical disability] and the number of people experiencing any other ‘non-serious’ adverse event [defined as any other unfavourable or unintended diagnosis, sign, symptom or disease]).

## Statistical analysis

IBM SPSS Statistics version 29.0.0.0 (241) was used for all analyses. Data was reported as mean and standard deviation. All randomized participants were included in the analysis (intention-to-treat).^24^ For repeated measurements linear mixed model was used. The between-group difference in the primary outcome (Victorian Institute of Sports Assessment – Achilles questionnaire) at 12 weeks was estimated at each timepoint using a repeated-measures linear mixed-effects regression model. The model included two predictor terms (time, baseline, 6 and 12 weeks; and group, radial extracorporeal shockwave therapy versus sham) and was adjusted for the fixed effects of age, sex, body mass index and baseline scores on the outcomes. Random effects were the participant-level variance. The main effect of interest was the interaction between time and group. Statistical tests were two-tailed with statistical significance set at 5% and corresponding 95% confidence intervals reported. Post hoc comparisons were conducted to explore differences at specific timepoints, without applying Bonferroni adjustments. An identical procedure was followed to estimate between-group differences for all secondary outcomes.

## Results

**Figure 2.**
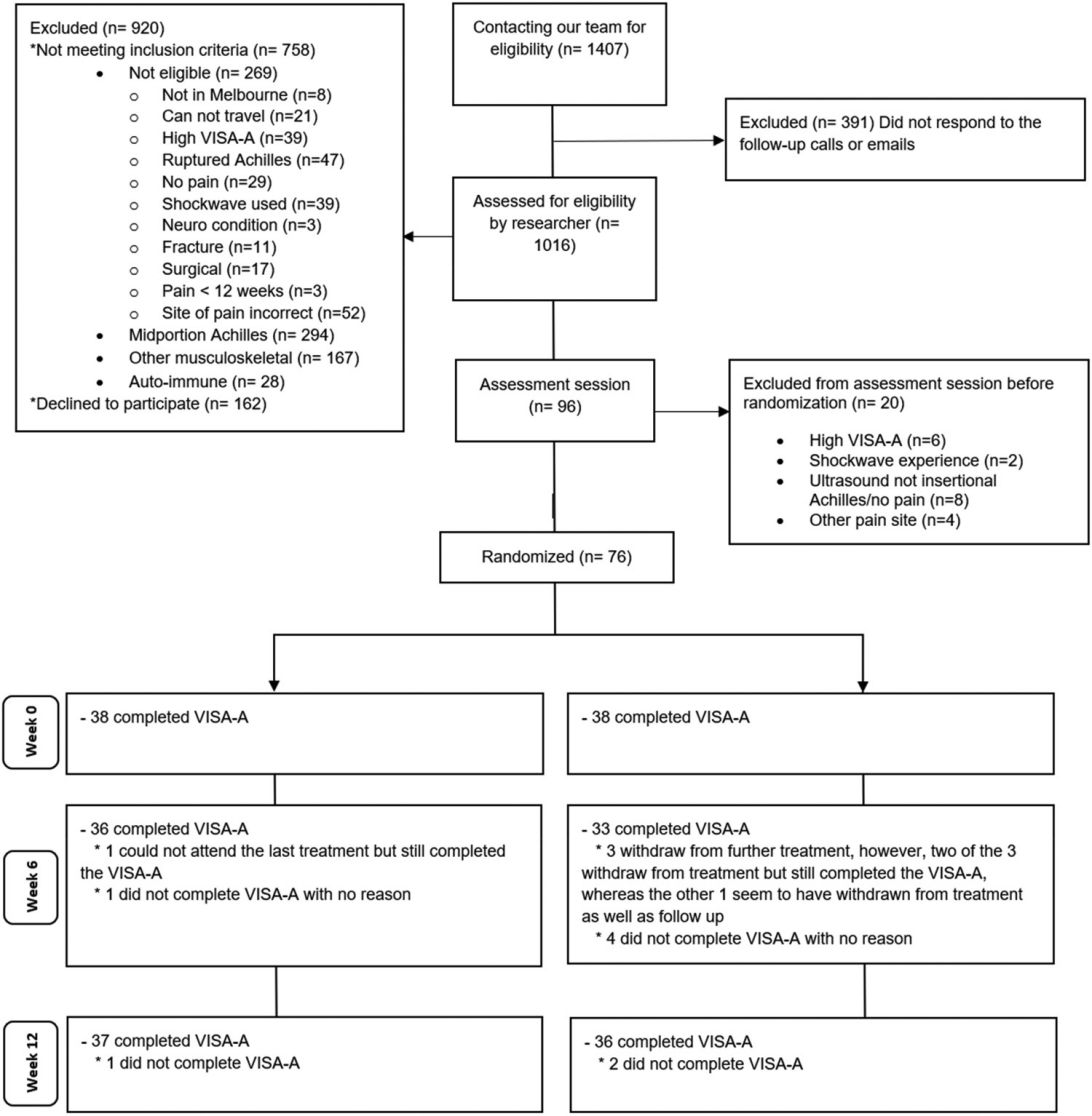
CONSORT flow diagram.*VISA-A = Victorian Institute of Sports Assessment – Achilles questionnaire*.

Between April 2021 and November 2022, we assessed 1407 participants for eligibility, of whom 96 (6.8%) were eligible (Figure 2). A total of 76 (79.2%) agreed to participate in the trial and were randomised to receive either radial extracorporeal shockwave therapy (*n* = 38 [50%]) or sham (*n* = 38 [50%]) (Figure 2). Seventy-six participants (100%) completed the Victorian Institute of Sports Assessment – Achilles questionnaire at baseline. There were three participants (8%) in the sham group who did not attend the second and third treatment sessions and one participant (3%) in the radial extracorporeal shockwave therapy who did not attend the third treatment session. Follow-up Victorian Institute of Sports Assessment – Achilles questionnaire outcome data were obtained from 69 participants (36 in radial extracorporeal shockwave therapy and 33 in sham, 91%) at 6 weeks and from 73 participants (37 in radial extracorporeal shockwave therapy and 36 in sham, 96%) at 12 weeks (Figure 2). At the end of the first treatment, the proportion of people in the sham (94.8) and intervention groups (100%) that thought they were receiving the intervention or were not sure were comparable.

All participants received their allocated treatment (radial extracorporeal shockwave therapy or sham). Table 1 shows baseline participant characteristics for each group. Body mass index was slightly higher in the sham group (*p* = .05), but the groups were well-matched in terms of other demographic data and severity. Mean age was between 48 and 52 years.

**Table 1.** Mean (standard deviation) of demographic and baseline outcomes data.

	Radial extracorporeal shockwave therapy mean	Sham mean
Age (year)	48.5 (12.2)	52.5 (11.1)
Sex: female/male (%)	19/19 (50%)	21/17 (55%)
Weight (kg)	88 (24.7)	94.5 (18.7)
Height (cm)	172.5 (10.1)	172.3 (10.3)
Body mass index (kg/m^2^)	29.4 (5.9)	31.9 (4.8)
Symptom duration (months)	33.3 (44.1)	47.8 (71)
Walking/running	16/22	22/16
Victorian Institute of Sports Assessment – Achilles questionnaire	46.3 (13.6)	47.7 (16.2)
Worst pain last 24 hours	3.1 (1.6)	3.7 (1.8)
7-day Recall Physical Activity Questionnaire-mets	246.1 (81)	283.3 (193.01)
Tampa Scale of Kinesiophobia	25.5 (12.9)	28.5 (28.1)
Pain Catastrophising Scale	14.5 (8)	15.3 (13)
Pain Self-Efficacy Questionnnaire	46.8 (10.3)	46.1 (11.2)
EuroQol-5D-5L	0.81 (0.2)	0.78 (0.2)
EuroQol-5D-5L – visual analogue scale	72.1 (15.7)	71.1 (18.5)

*Significant.

**Figure 3.**
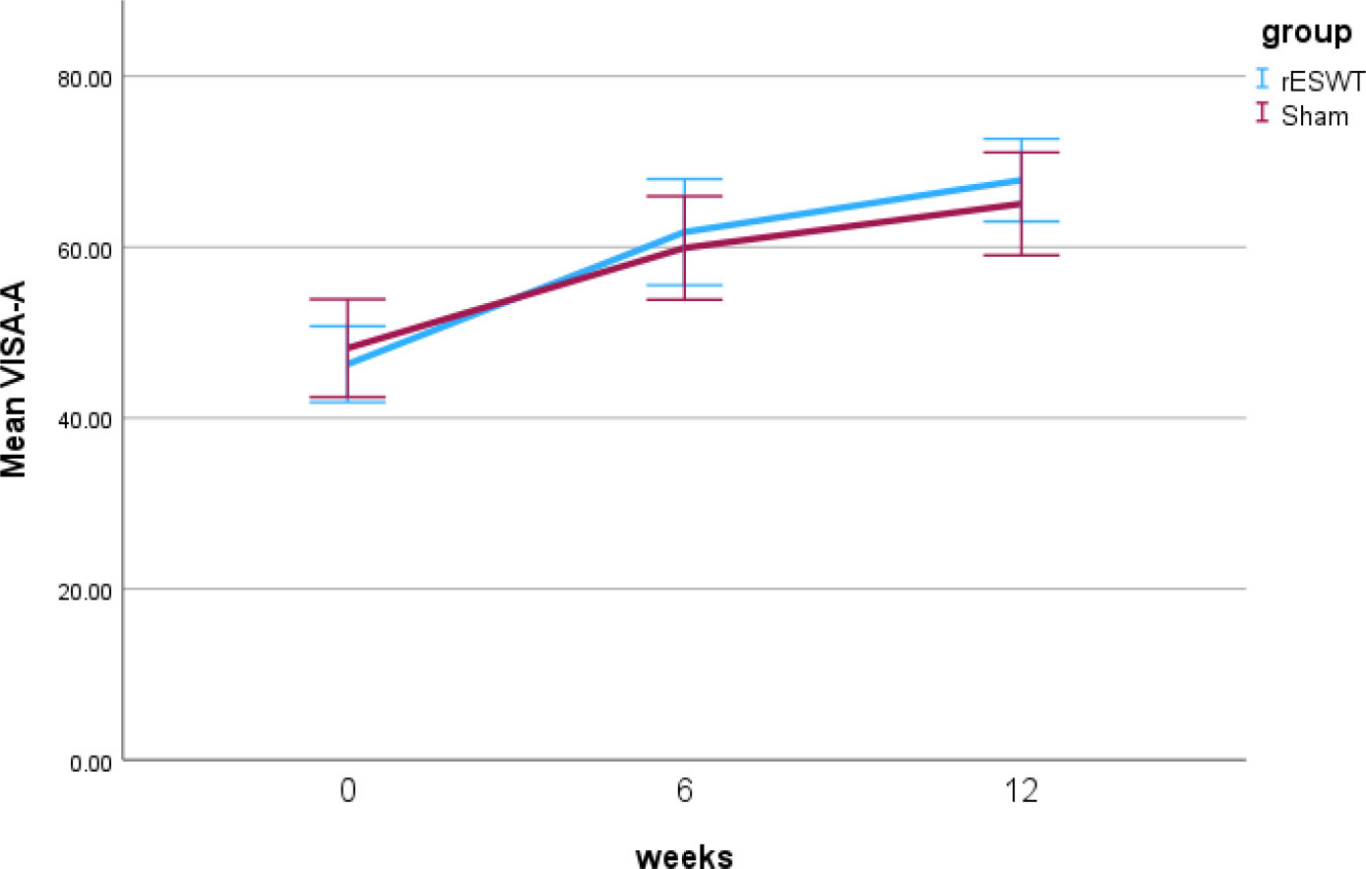
The two-way interactions (radial extracorporeal shockwave therapy [rESWT] versus sham by weeks) for the Victorian Institute of Sports Assessment – Achilles (VISA-A) questionnaire.

The adjusted results for the primary and secondary outcomes are presented in Table 2 (the unadjusted analysis is presented in Supplementary File 3). There were no between-group differences in Victorian Institute of Sports Assessment – Achilles questionnaire scores at 6 weeks (3, 95% confidence interval −4.6–10.5, *p* = .44) or 12 weeks (4.6, 95% confidence interval −2.5–11.6, *p* = .20) (Figure 3 and Table 2). Likewise, there were no significant differences for secondary outcomes (*p* > .05) (see Table 2).

**Table 2.** Adjusted analysis for primary and secondary outcome measures mean (standard deviation) and interactions.

Outcome	Week	Radial extracorporeal shockwave therapy	Sham	Between-group adjusted difference Coefficient (95% confidence interval)^$^	*p* Value
Victorian Institute of Sports Assessment – Achilles questionnaire	0	44.8 (15.1)	49.4 (15.1)		
	6	59.9 (15.4)	60.6 (16.2)	−0.7 (−7.9–6.6)	.86
	12	66.4 (15.2)	64.6 (15.2)	1.8 (−5.2–8.7)	.62
Worst pain last 24 hours	0	3.1 (2)	3.7 (2.1)		
	6	2.7 (2.1)	3.2 (2.2)	−0.5 (−1.5–0.5)	.29
	12	2.8 (2.3)	2.9 (2.4)	−0.1 (−1.2–1)	.87
7-day Recall Physical Activity Questionnaire-mets	0	246.5 (126.5)	283.2 (126.8)		
	6	259 (128.8)	227.9 (136.2)	31.1 (−29.9–92.1)	.32
	12	260.4 (119.5)	251.6 (120.9)	8.8 (−46.3–64)	.75
Tampa Scale of Kinesiophobia	0	25.3 (16.4)	28.9 (16.4)		
	6	23.2 (16.4)	25.1 (17.3)	−1.9 (−9.6–5.8)	.63
	12	23.9 (16.3)	23.3 (16.1)	0.6 (−6.8–8.1)	.87
Pain Catastrophising Scale	0	14.7 (10.9)	15.1 (10.3)		
	6	9.5 (10.4)	6.9 (10.8)	2.6 (−2.3–7.6)	.29
	12	8.3 (10.2)	7.7 (10.2)	0.6 (−4.1–5.3)	.80
Pain Self-Efficacy Questionnnaire	0	46.6 (9.8)	46.3 (9.9)		
	6	51.3 (10)	54 (10.4)	−2.7 (−7.3–2)	.26
	12	52.7 (10.7)	51 (10.7)	1.7 (−3.2–6.6)	.50
EuroQol-5D-5L	0	0.81 (0.1)	0.78 (0.1)		
	6	0.88 (0.1)	0.87 (0.1)	0.01 (-−0.06–0.08)	.74
	12	0.91 (0.1)	0.89 (0.1)	0.02 (−0.05–0.08)	.56
EuroQol-5D-5L visual analogue scale	0	71.7 (17.4)	71.4 (17.4)		
	6	77.6 (17.6)	82.1 (18.4)	−4.5 (−12.8–3.8)	.28
	12	81.2 (17.7)	77.1 (17.5)	4 (−4.1–12.2)	.33
Productivity Cost Questionnaire_presenteesim	0	−	−		
	6	3295.1 (11,899.2)	−201.8 (11,899.2)		
	12	2343.5 (11,899.2)	2177.7 (11,899.2)	165.8 (−5278.2–5609.8)	.95
Productivity Cost Questionnaire _unpaid	0	−	−		
	6	408.5 (904.7)	2.7 (904.7)		
	12	439.2 (904.7)	−18.8 (904.7)	458 (44.1–817.2)	.03

*Age, sex and body mass index, ^$^Comparisons made to baseline.

Table 3 shows the bar pressure achieved in the radial extracorporeal shockwave therapy ranges from 1.0 to 1.7 over the three sessions. The mean of maximal pain reported during the radial extracorporeal shockwave therapy treatment was higher (7.6 [1.1]) than the maximum pain during the sham sessions (3.8 [2.5]) (Table 3).

**Table 3.** Pain and treatment pressure during sessions.

Session	Pain − mean (standard deviation) (adjusted 95% confidence interval)		
	Radial extracorporeal shockwave therapy	Sham	Significant difference (*p* < .05)
First	7.6 (1.1) (7.0–8.3)	3.8 (2.5) (3.2–4.4)	.00
Second	7.3 (1.2) (6.8–7.9)	3.1 (1.8) (2.5–3.6)	.03
Third	7.0 (1.6) (6.4–7.6)	2.8 (1.8) (2.2–3.4)	.03
	Pressure Median (interquartile range)		
First	1.0 (0.7–1.8)	5.0 (4.0–5.0)	−
Second	1.3 (1–1.3)	5.0 (5.0–5.0)	−
Third	1.7 (1.8–2.6)	5.0 (5.0–5.0)	−

No serious adverse events were reported (Table 4). The number of people experiencing a non-serious adverse was higher in the radial extracorporeal shockwave therapy group, but only in the second 6-week period of observation (Table 4). Non-serious adverse events included Achilles pain after the treatment session lasting up to 2 days, pain at the Achilles related to the exercises prescribed or excessive walking or running, and sports involvement (Supplementary File 4). Use of other treatments was also similar between the groups (Table 4). Exercise adherence was similar in both groups at 6 (radial extracorporeal shockwave therapy = 68%; Sham = 65%, *p* = 1.00) but higher in the radial extracorporeal shockwave therapy group at 12 weeks (radial extracorporeal shockwave therapy = 67%; Sham = 49%, *p* = .80) (Table 4). 

**Table 4.** Adverse events, other treatment seeking during the trial period and exercise adherence.

	Week	Radial extracorporeal shockwave therapy	Sham	*p* Value*
Number of non-serious adverse events	6	9 (23.7%)	9 (23.7%)	.89
	12	15 (39.5%)	6 (15.8%)	.02
Number of non-serious adverse events related to the treatments	6	1 (0.03%)	2 (0.05%)	
	12	0 (0%)	0 (0%)	
Number of people seeking other treatment	6	4 (10.5%)	4 (10.5%)	1.00
	12	3 (7.8%)	3 (7.8%)	1.00
Number of treatment sessions with a health professional	6	0 (0 session)	4 (9 sessions)	
	12	6 (17 sessions)	6 (16 sessions)	

* Chi−square.

## Discussion

This trial addresses an important research gap by investigating the efficacy of radial extracorporeal shockwave therapy in combination with recommended care in people with insertional Achilles tendinopathy. The exercise program included continued walking and sport activity dependent on individual pain levels. This approach is currently recommended for insertional Achilles tendinopathy rehabilitation.^1,4^ Despite using a recommended exercise approach, we did not find any evidence to support the addition of radial extracorporeal shockwave therapy compared to sham. There were no between-group differences for our primary composite pain, participation and disability outcome at 6 (3, 95% confidence interval −4.6–10.5) or 12 weeks (4.6, 95% confidence interval −2.5–11.6). There was also no evidence of a between-group difference for any secondary outcome measures at either 6 or 12 weeks (*p* > .05). No serious adverse events were reported.

There were no between-group differences, indicating that the addition of radial extracorporeal shockwave therapy was not superior to sham. There are two possible explanations for our findings. First, the addition of radial extracorporeal shockwave therapy did not add any benefit over and above the exercise and education treatments or natural history. Second, there may have been some benefit from radial extracorporeal shockwave therapy, but this was explained by a placebo effect and therefore similar in the sham group. It is known that the placebo response may be influenced by the therapy being offered, patient-clinician interaction and the clinician's personality (e.g. the clinician's assurance in the therapy and description of the course of treatment) and contextual factors such as the clinic's standing and reputation.^25^ Past experiences (either positive or negative) can also influence outcomes, but we controlled for these by only including people who had not previously trialled radial extracorporeal shockwave therapy. To further investigate whether the benefit in our trial was due to placebo or some effect of exercise, trials are needed to investigate the effect of exercise versus sham or wait-and-see.

Two other studies have investigated radial extracorporeal shockwave therapy with placebo comparators among people with insertional Achilles tendinopathy. Mansur et al.^6^ investigated the efficacy of adding radial extracorporeal shockwave therapy or sham to exercise for insertional Achilles tendinopathy. They prescribed an eccentric training program modified so that it is performed on flat ground (avoiding full dorsiflexion). The isolated eccentric training program aims to reproduce moderate to severe pain during the exercise; otherwise, more load is added and it is performed twice per day, 7 days per week.^6^ Mansur et al.^6^ also advised people to stop all sport activity for 8 weeks regardless of their symptoms, which is not recommended based on current evidence.^4^ In this trial, participants were allowed to maintain and even progress with running and sporting activity depending on pain levels. However, similar to the study by Mansur et al.^6^, we found no between-group differences in primary pain and function outcomes at any time point (2, 4, 6, 12, 24 weeks for Mansur and 6 and 12 weeks in our study). The mean age (53 years old in Mansur et al.^6^ and 50 years old in our study) and sex (48% females in Mansur et al.^6^ and 53% females in our study) were similar between the studies. It is also interesting to note that within-group improvements in our study (mean Victorian Institute of Sports Assessment – Achilles questionnaire baseline = 47; mean Victorian Institute of Sports Assessment – Achilles questionnaire 12 weeks = 61) and Mansur et al.^6^ (mean Victorian Institute of Sports Assessment – Achilles questionnaire baseline = 42; mean Victorian Institute of Sports Assessment – Achilles questionnaire 12 weeks = 58) indicate similar benefits from different exercise programs, although this may be explained by non-specific effects, such as natural history or regression to the mean. Pinitkwamdee et al. investigated conventional treatment (rest, medication, activity modification, stretching exercise, and heel lift orthosis) with the addition of radial extracorporeal shockwave therapy or sham.^26^ The stretching exercise was not well defined, and the study was underpowered (a total of 31 people randomised to the two groups). There were no between-group differences in self-reported pain (composite pain and function were not reported) at any timepoint. Overall, there is no current evidence to support the use of radial extracorporeal shockwave therapy when compared with sham for insertional Achilles tendinopathy, regardless of adjunct therapies or exercise programs.

A strength of this trial is that it was adequately powered (76 participants), and participants and the outcome assessors were masked to treatment allocation. There were limitations to our study that we need to highlight. First, although pain was significantly lower in the sham group compared with the radial extracorporeal shockwave therapy group, there was still substantial pain (mean pain during treatment 7.31 (0.33) for sham versus 3.22 (0.51) for treatment groups) during the sham treatment. The pain in the sham group is likely to be produced by holding the applicator against the posterior calcaneum which is commonly tender and painful to palpate among people with insertional Achilles tendinopathy.^1^ The reason pain in the sham arm is a limitation is that one of the proposed (although not established) mechanisms of radial extracorporeal shockwave therapy is conditioned pain modulation (endogenous pain inhibition following a painful stimulus).^27^ Future research is needed using different shams that do not elicit pain. Second, the physiotherapists applying the radial extracorporeal shockwave therapy treatment were not masked to treatment allocation. We attempted to mitigate this risk of experimenter bias by scripting, as much as possible, the interactions between these physiotherapists and the participants. Third, the level of energy that was delivered to the insertion of the Achilles tendon is not certain. Radial extracorporeal shockwave therapy intensity is based on energy flux density, with values of <0.08 MJ/mm^2^, < 0.28 MJ/mm^2^, and <0.60 MJ/mm^2^ representing low, medium and high intensities, respectively.^28^ According to the manufacturer's report, the dose we selected (10 Hz, 3000 shocks, 2­–4 bar) is likely to deliver approximately 0.63 MJ/mm^2^ to the region of interest with the machine we used.^29,30^ What is not known is the minimum dose required for an effect, and this has been debated in the literature.^31^

The addition of radial extracorporeal shockwave therapy to recommended exercise and education did not result in improved pain and function or other outcomes compared to sham at 6 or 12 weeks among people with insertional Achilles tendinopathy.

**Clinical messages**

- Clinician's may consider radial extracorporeal shockwave therapy for the management of insertional Achilles tendinopathy, but any benefits found may be explained by the placebo mechanism.
- Radial extracorporeal shockwave therapy is relatively safe, with few minor adverse events and no serious adverse events were found in this trial.

## Supplemental Material

sj-docx-1-cre-10.1177_02692155241295683 - Supplemental material for Does shockwave therapy lead to better pain and function than sham over 12 weeks in people with insertional Achilles tendinopathy? A randomised controlled trial Supplemental material, sj-docx-1-cre-10.1177_02692155241295683 for Does shockwave therapy lead to better pain and function than sham over 12 weeks in people with insertional Achilles tendinopathy? A randomised controlled trial by Baraa Alsulaimani, Luke Perraton, Patrick Vallance, Tim Powers and Peter Malliaras in Clinical Rehabilitation
