# Supplementary material for: Does shockwave therapy lead to better pain and function than sham over 12 weeks in people with insertional Achilles tendinopathy? A randomised controlled trial
Source: Clin Rehabil. 2024 Dec 20;39(2):174–86. doi: 10.1177/02692155241295683 (PMC11846266; doi:10.1177/02692155241295683)
Supplement: sj-docx-1-cre-10.1177_02692155241295683 - Supplemental material for Does shockwave therapy lead to better pain and function than sham over 12 weeks in people with insertional Achilles tendinopathy? A randomised controlled trial [file sj-docx-1-cre-10.1177_02692155241295683.docx]

**Supplementary file one. Education sheet**

Achilles Tendinopathy General Information

- Achilles tendon pain is common
- Change in activities that load the Achilles tendon is the most common cause
- Advice and exercise are the most important treatments, but some people require other treatments like shockwave therapy.

**The Achilles tendon**

| A tendon attaches muscles to bone. Your Achilles tendon is found at the back of the lower leg, just above the heel bone. It attaches your two calf muscles (gastrocnemius and soleus) to the heel bone (calcaneus) and helps you go up onto tiptoes.  The Achilles tendon is the biggest and strongest tendon in the body. Its main role is to transfer load from muscle to bone. | 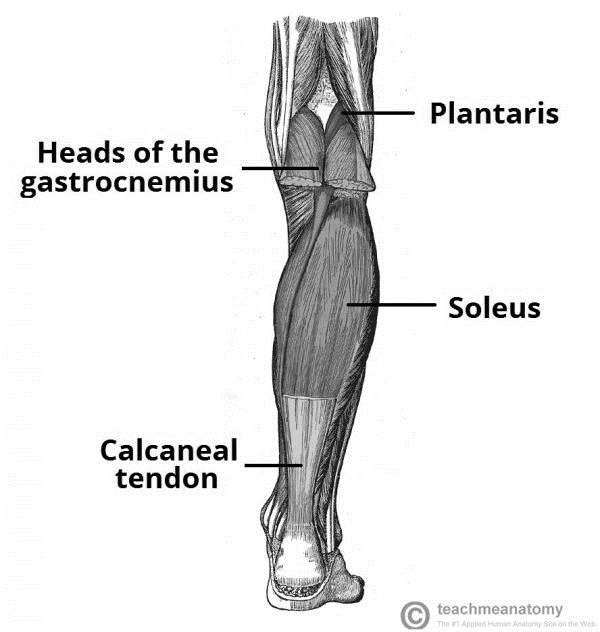 |
| --- | --- |

**What is Achilles tendinopathy?**

This condition is characterised by pain in the Achilles tendon, previously referred to as Achilles tendinitis. There are two types of Achilles tendinopathy: Mid-portion, where pain is usually present in the middle portion of the Achilles tendon; and insertional, which affects the spot where the tendon meets the heel bone. There are many risk factors for Achilles tendinopathy, including:

- **Changes in activity** – this occurs when new activities surpass the capacity of the tendon. For example, when starting to walk or run more, or starting to run up hills more. Athletes can develop Achilles tendinopathy, but it is also a common overuse condition in people not involved in sport.
- **Older age**
- **Metabolic issues** e.g. elevated cholesterol or diabetes.
- **Overweight and obesity** – being overweight places more strain on the Achilles tendon.
- **Onset of menopause** in females

**Common symptoms of Achilles tendinopathy**

- **Morning stiffness:** Many people complain of stiffness around the tendon when they get up in the morning. This usually eases after a few minutes of walking, but may last longer.
- **Tenderness over the Achilles tendon:** Often the tendon is very tender to touch when gently squeezed. There may be a tender lump in the tendon.
- **Pain with exercise:** Pain during or after weight-bearing activity (such as walking, running and jumping) is common. For some people, the pain improves with activity. Others experience severe pain in their Achilles tendon which stops them from doing their activity.

**How is Achilles tendinopathy diagnosed?**

It can be diagnosed by health care professionals who treat Achilles tendon pain such as a sports doctor, general practitioner, physiotherapist or podiatrist. Diagnosis is based on asking questions to identify the potential cause and factors that aggravate your pain, for example, type and frequency of exercises etc. A physical examination will be required as well as testing your movements and leg strength.

**Do I need a scan?**

We don’t always need to carry out X-rays or scans (imaging) to diagnose Achilles tendinopathy. However, an ultrasound scan may sometimes be needed if the diagnosis is not clear. Ultrasound is a quick, safe and effective way for us to see your tendon. Magnetic Resonance Imaging (MRI) may also be used, but this is not common.

**What are the treatments for Achilles tendinopathy?**

***The first aim of the treatment*** is to reduce pain. This can be achieved by:

- Avoiding or limiting activities that may aggravate the condition, such as excessive walking and running. This can be replaced with activities that do risk aggravating Achilles pain such as swimming or cycling.
- Applying ice to the tendon
- Gentle exercises

***The second aim of treatment*** is to perform specific exercises that strengthen the tendon and associated muscles and this may also help your pain.

***The third aim of treatment*** is to gradual resume activities that you have had to reduce due to your pain. This involves starting with a few minutes and increasing the time spent doing these activities over a number of weeks. Monitor your pain during and after exercises. Do the activity with less than 5/10 pain. If you experience more pain, then reduce the activity by 50% or stop completely, and resume when the pain is improved. We will guide you during this process.

**What about medications?**

If you feel you really need more pain cover, you could take paracetamol 500mg. See you doctor if you are not sure whether you are able to take this.

**Prognosis**

Since the tendon weakening (degeneration) usually occurs over months or years prior to onset of symptoms; recovery may take a few months. Most people experience significant improvement in their Achilles tendon pain and ability to perform their normal activities with the right advice and exercise over **a period of 12 weeks**.

**Why is exercise recommended if it was the cause of my Achilles problem?**

People with Achilles tendinopathy sometimes find it strange that exercise is recommended to treat Achilles tendon pain when too much activity often causes it. However, introducing appropriate exercises gradually is a very effective way to increase the capacity of your Achilles tendon by making the muscles and tendon stronger and more resistant to loading. Please refer to the exercise booklet for further details on the type of exercises and how to progress gradually.

**But it hurts! Is it normal to feel pain during activity and exercise for my Achilles tendon pain?**

Some people avoid exercises completely out of fear they will cause further damage. It is common and in fact quite normal to feel some pain when performing or after performing exercises. This pain usually settles within a reasonable period of time, less than a day. Some pain with exercise is NOT a sign of damage.


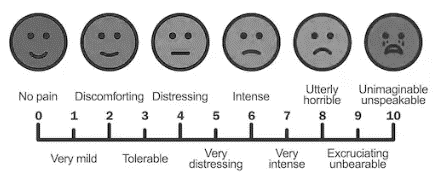
If you feel an activity or exercise is too painful, stop doing the exercise or modify how you do it.

‘Too painful’ is defined as equal to or more than 5/10 pain. When your pain settles again, it is safe and suggested to recommence the activity or the exercise.

**Other treatments like shockwave and surgery**

Treatments like shockwave are usually considered if there’s no change in symptoms after trying the above suggested treatments. Other options like injection may also be helpful. Your treating practitioner will discuss these options with you.

Surgery is the last resort if other treatments have failed. It is not guaranteed to relieve your symptoms.

**Supplementary file two. Exercise sheet**

**EXERCISE INFORMATION**

As a participant of this research study you are expected to perform an exercise training program. There are some important guidelines to observe whilst performing the exercises:

You will need to perform the exercises on both legs alternatively as per the instructions below:

**TEMPO:**

3 seconds up, 3 seconds down

**FREQUENCY:**

3x/week

**REPETITIONS:** 15 repetitions x 4 sets (with 2 minutes rest between sets and 1 minute between Exercise A and Exercise B)

**It is very important to note that during this exercise program you may experience some pain and/or fatigue.** Continue the exercise even if you experience pain up to 5 out of 10, based on the pain scale below. The pain will reduce as you continue your rehabilitation.

**PAIN SCALE**


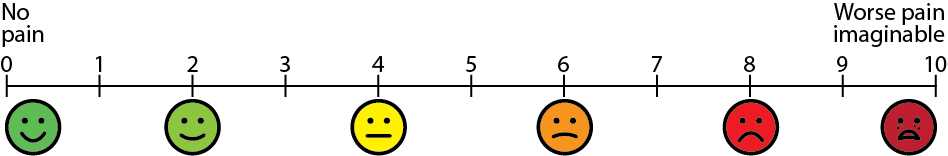


To help you keep track of these exercises and your symptoms, record your progress at the end of this document.

1.
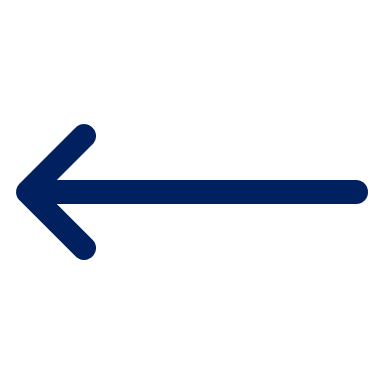
**Exercise A: Calf raise with straight knee**

**Knee straight: Level 2**

**Knee straight: Level 1**

Stand upright on a flat surface next to a wall. Use the wall to balance yourself if required.

Standing on one leg, lift the heel **as high as possible** on your weight bearing leg, rising up onto your toes.

Slowly lower the heel down again

Make sure you **keep the knee straight** throughout the exercise

**Level 2:** Add 5kg weight to increase the load on the tendon, either in one hand or back-pack (rice packets, books, bricks etc).

Continue increasing in 5kg


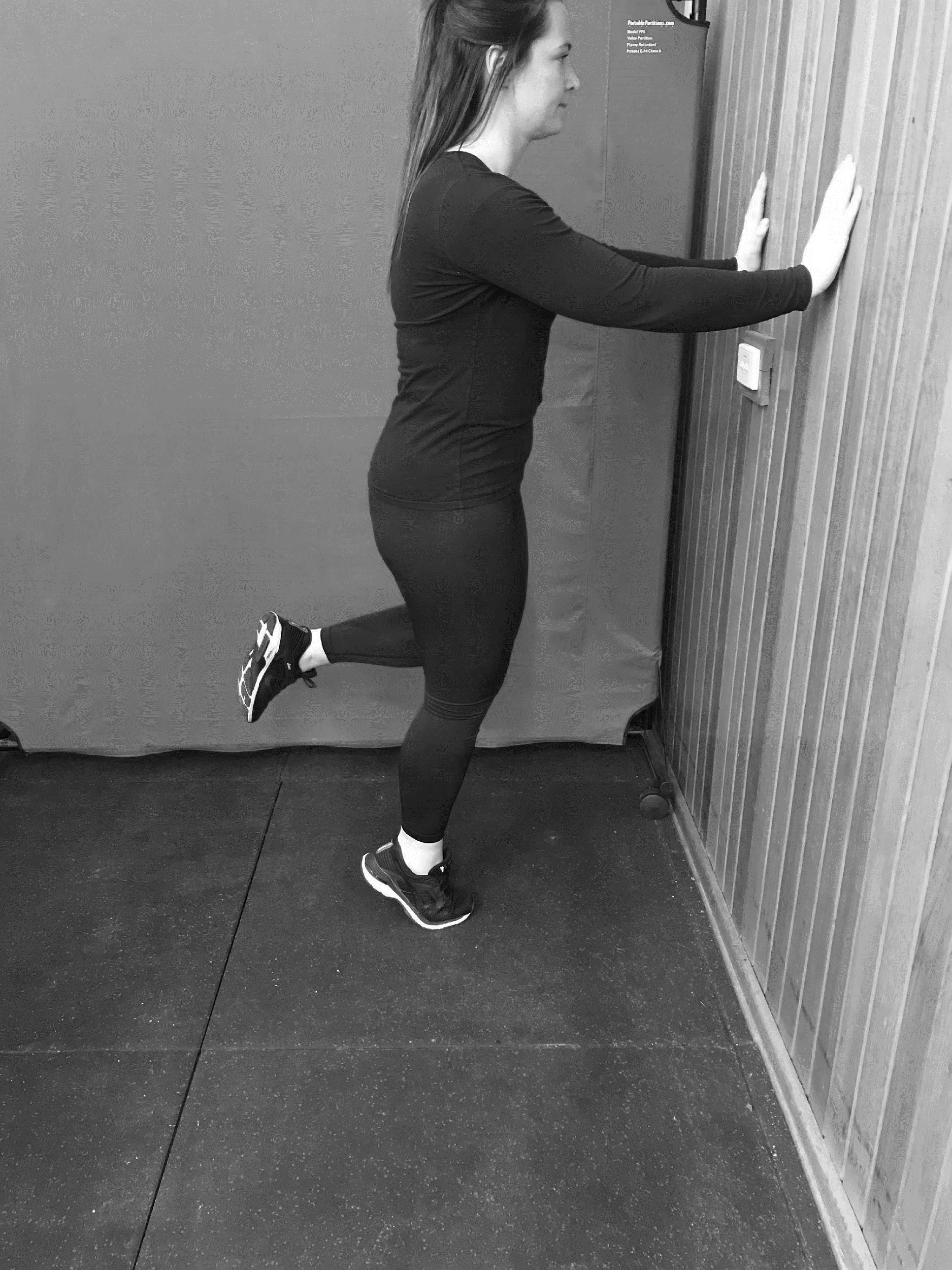

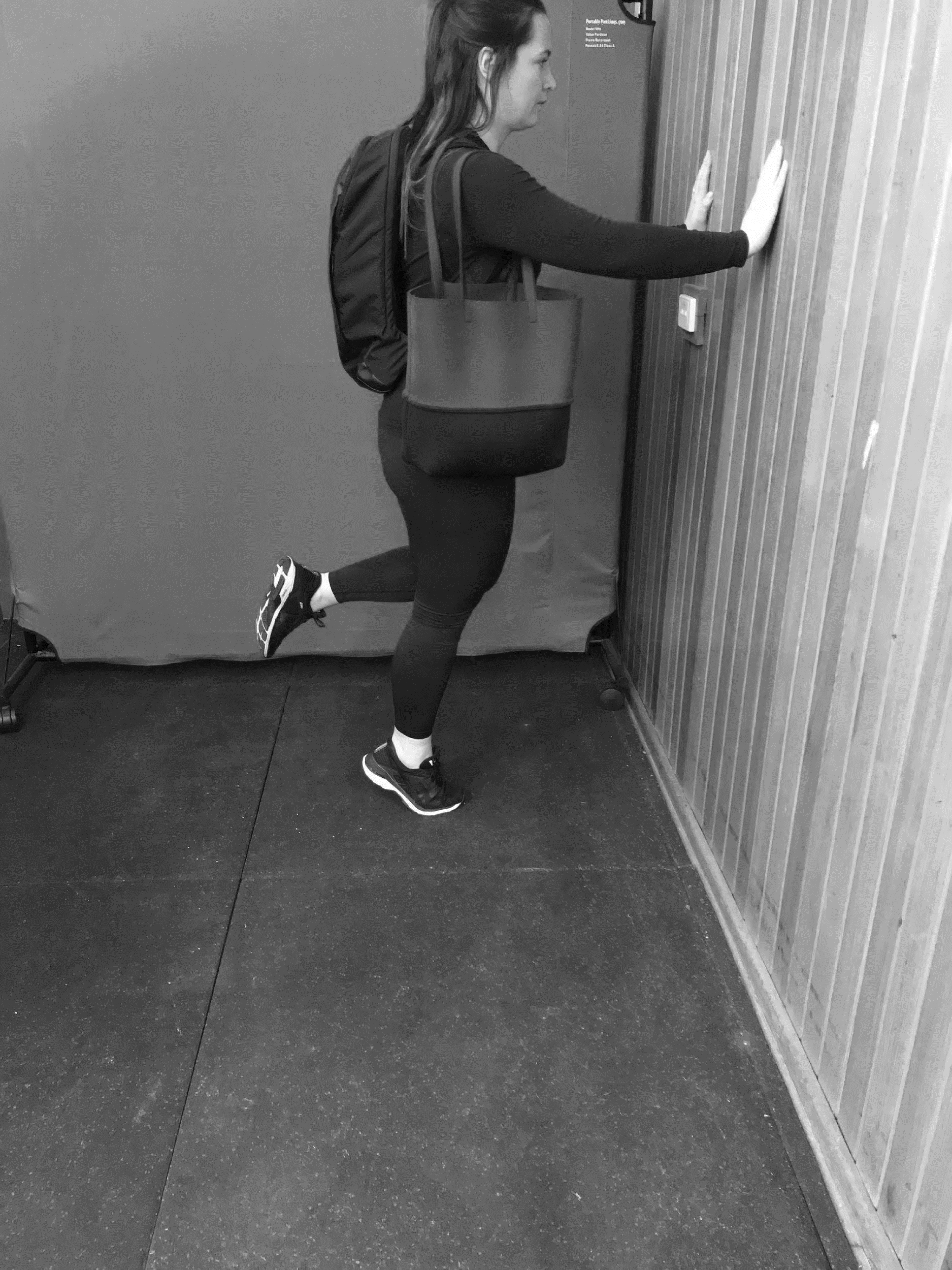


| **Is this exercise too easy or difficult?** |
| --- |
| Refer to the modifications table for this exercise and follow the steps to make it more suitable for you. |

1. **Exercise B: Calf raise with bent knee**

Stand leaning forward on a wall. Bend knee to approximately 20-30 degrees of bend.

Standing on one leg, lift the heel **as high as possible** on your weight bearing leg

Slowly lower the heel down again

Make sure you **keep the knee bent** throughout the entire movement

**Level 2:** Add 5kg weight to increase the load on the tendon, either in one hand or back-pack (rice packets, books, bricks etc). Continue increasing in 5kg increments as tolerated.

**Knee bent: Level 1**

**Knee bent: Level 2**


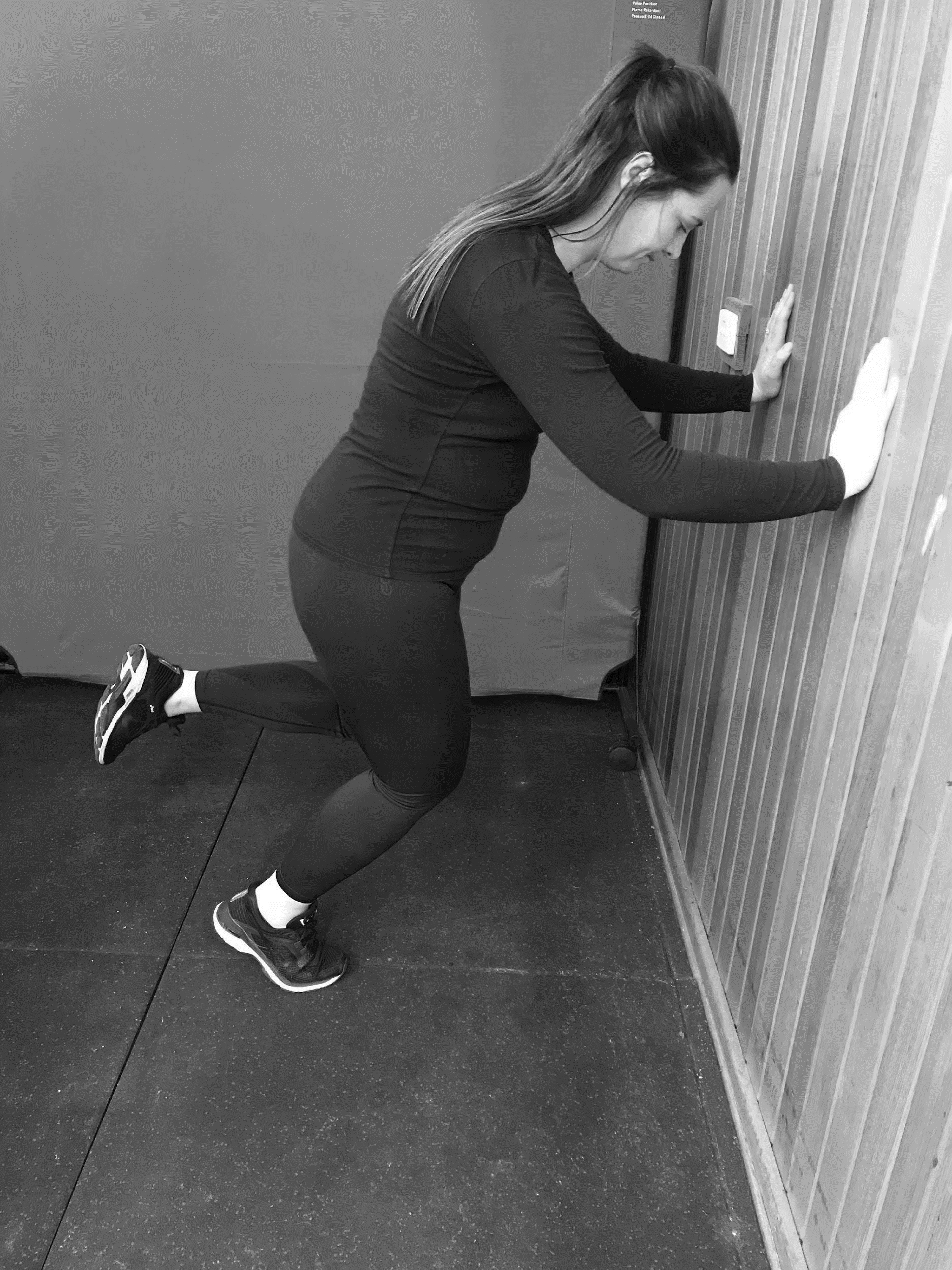

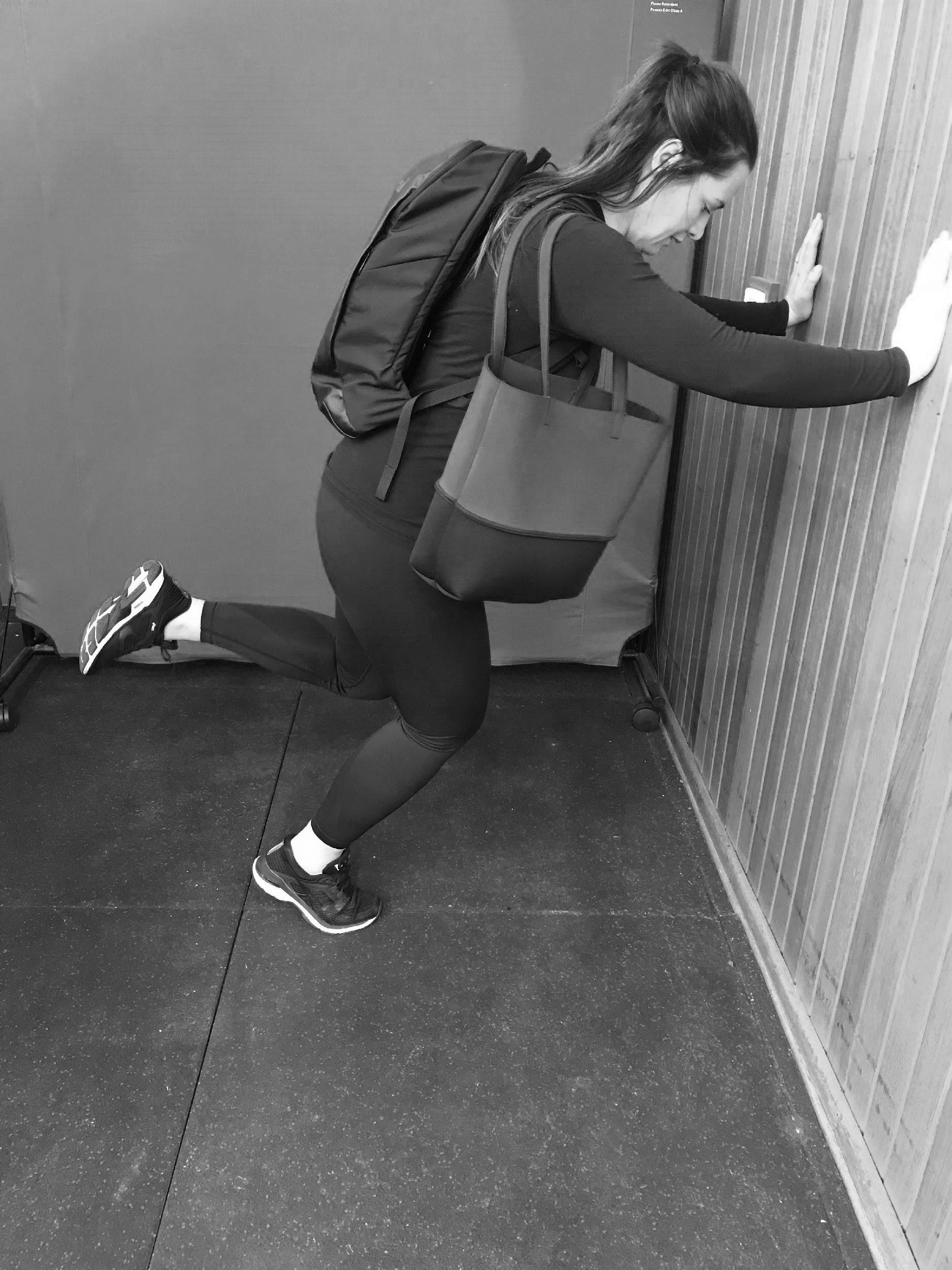


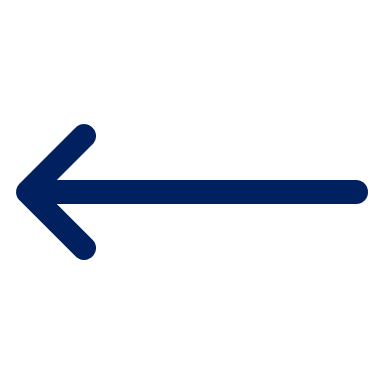


| **Is this exercise too easy or difficult?** |
| --- |
| Refer to the modifications table for this exercise and follow the steps to make it more suitable for you. |

**Modification table**


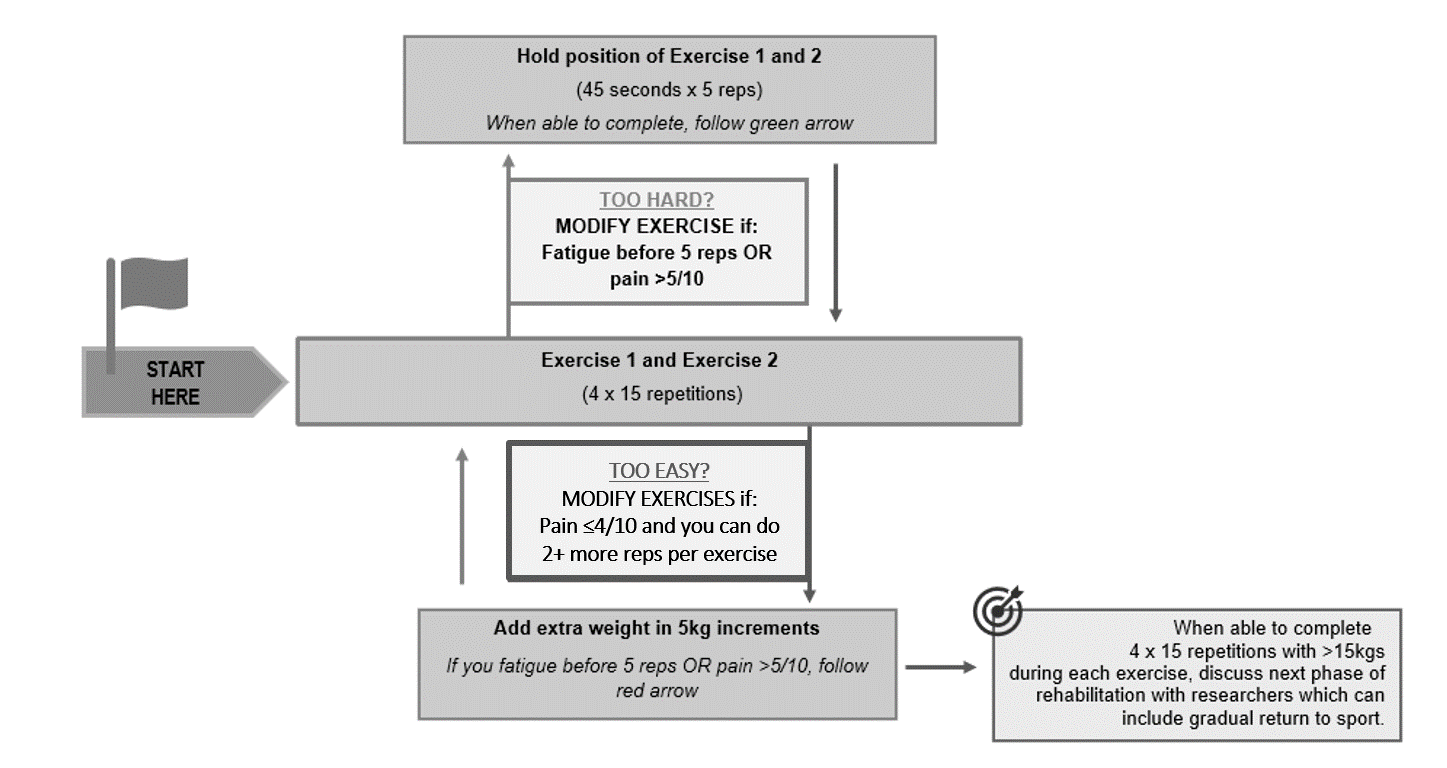


**Education and exercise diagram
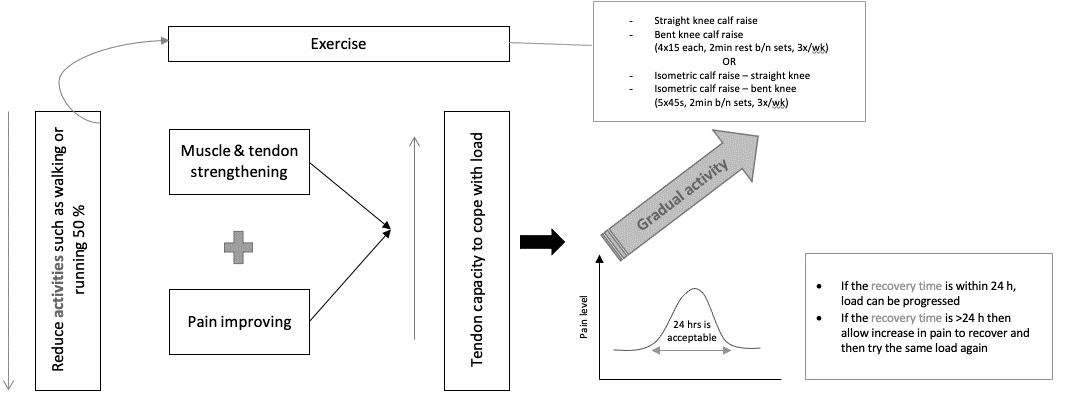
**

**How to progress and regress your exercise**

| **LOAD TEST (do 4-5 times)** | **Pain ‘acceptable’ = ≤4/10** | **Pain 5-7/10** | **Pain ≤8/10** |
| --- | --- | --- | --- |
| IF YOU ARE A WALKER:  5 x 1 leg calf raise | - Isotonic is ok - If you can do 4 or more reps at the end add 5kg - Add a maximum of 5kg per week - Your goal should be 10-15kg (walkers) or 20-25kg (runners) | - Regress to isometric - Keep adding 5kg per week if you are finding you can do it easily - Also reduce walking, running, running sports by 50% - Stop any intensity (e.g. hill or fast walking/running) | - Regress to isometric - Also reduce walking to only incidental and stop all running/running sports |
| IF YOU ARE A RUNNER:  5 x 1 leg calf raise  AND  5 x 1 leg continuous hops |  |  |  |
| Progressing walking/running | - Not for the first 2 weeks | - Test load tests every session to see if you are ready to progress | Test load tests every session to see if you are ready to progress |

**A guide to progressing walking/running**

|  | 10% | 20% |
| --- | --- | --- |
| Full time/ minutes | **Minutes** | **Minutes** |
| 10 | 1 | 2 |
| 20 | 2 | 4 |
| 30 | 3 | 6 |
| 30 | 3 | 6 |
| 40 | 4 | 8 |
| 60 | 6 | 12 |

**MY GOALS**

In this section please write down your weight goals. An example might be to be able to perform 4 sets of 15 of the calf raise exercise with 30kg by the end of the program (12 weeks).

**Exercise adherence**

In this section, please keep a record of the exercises you have done. For example:

| **Date**  **1** | **Exercises / Repetitions / Weight** | | | | | | |
| --- | --- | --- | --- | --- | --- | --- | --- |
|  | **Exercise 1 (with knee straight)** | Did you do the prescribed 4 sets of 15 repetitions? | Exercise weight (kg) | Step added | **Exercise 2 (with knee bent)** | Did you do the prescribed 4 sets of 15 repetitions? | Exercise weight (kg) |
| **Date**  **2** | Yes/No | Yes/No  If no, how many______  why? __________________________ |  | Yes/No | Yes/No | Yes/No  If no, how many______  Why? ______________________ |  |
| **Date**  3 | Yes/No | Yes/No  If no, how many______  why? __________________________ |  | Yes/No | Yes/No | Yes/No  If no, how many______  Why? ______________________ |  |
| **Date**  **4** | Yes/No | Yes/No  If no, how many______  why? __________________________ |  | Yes/No | Yes/No | Yes/No  If no, how many______  Why? ______________________ |  |
| **Date** | Yes/No | Yes/No  If no, how many______  why? __________________________ |  | Yes/No | Yes/No | Yes/No  If no, how many______  Why? ______________________ |  |
| **Date** | Yes/No | Yes/No  If no, how many______  why? __________________________ |  | Yes/No | Yes/No | Yes/No  If no, how many______  Why? ______________________ |  |
| **Date** | Yes/No | Yes/No  If no, how many______  why? __________________________ |  | Yes/No | Yes/No | Yes/No  If no, how many______  Why? ______________________ |  |
| **Date** | Yes/No | Yes/No  If no, how many______  why? __________________________ |  | Yes/No | Yes/No | Yes/No  If no, how many______  Why? ______________________ |  |
| **Date** | Yes/No | Yes/No  If no, how many______  why? __________________________ |  | Yes/No | Yes/No | Yes/No  If no, how many______  Why? ______________________ |  |
| **Date** | Yes/No | Yes/No  If no, how many______  why? __________________________ |  | Yes/No | Yes/No | Yes/No  If no, how many______  Why? ______________________ |  |
| **Date** | Yes/No | Yes/No  If no, how many______  why? __________________________ |  | Yes/No | Yes/No | Yes/No  If no, how many______  Why? ______________________ |  |
| **Date** | Yes/No | Yes/No  If no, how many______  why? __________________________ |  | Yes/No | Yes/No | Yes/No  If no, how many______  Why? ______________________ |  |
| **Date** | Yes/No | Yes/No  If no, how many______  why? __________________________ |  | Yes/No | Yes/No | Yes/No  If no, how many______  Why? ______________________ |  |
| **Date** | Yes/No | Yes/No  If no, how many______  why? __________________________ |  | Yes/No | Yes/No | Yes/No  If no, how many______  Why? ______________________ |  |
| **Date** | Yes/No | Yes/No  If no, how many______  why? __________________________ |  | Yes/No | Yes/No | Yes/No  If no, how many______  Why? ______________________ |  |
| **Date** | Yes/No | Yes/No  If no, how many______  why? __________________________ |  | Yes/No | Yes/No | Yes/No  If no, how many______  Why? ______________________ |  |
| **Date** | Yes/No | Yes/No  If no, how many______  why? __________________________ |  | Yes/No | Yes/No | Yes/No  If no, how many______  Why? ______________________ |  |
| **Date** | Yes/No | Yes/No  If no, how many______  why? __________________________ |  | Yes/No | Yes/No | Yes/No  If no, how many______  Why? ______________________ |  |
| **Date** | Yes/No | Yes/No  If no, how many______  why? __________________________ |  | Yes/No | Yes/No | Yes/No  If no, how many______  Why? ______________________ |  |
| **Date** | Yes/No | Yes/No  If no, how many______  why? __________________________ |  | Yes/No | Yes/No | Yes/No  If no, how many______  Why? ______________________ |  |
| **Date** | Yes/No | Yes/No  If no, how many______  why? __________________________ |  | Yes/No | Yes/No | Yes/No  If no, how many______  Why? ______________________ |  |
| **Date** | Yes/No | Yes/No  If no, how many______  why? __________________________ |  | Yes/No | Yes/No | Yes/No  If no, how many______  Why? ______________________ |  |
| **Date** | Yes/No | Yes/No  If no, how many______  why? __________________________ |  | Yes/No | Yes/No | Yes/No  If no, how many______  Why? ______________________ |  |
| **Date** | Yes/No | Yes/No  If no, how many______  why? __________________________ |  | Yes/No | Yes/No | Yes/No  If no, how many______  Why? ______________________ |  |
| **Date** | Yes/No | Yes/No  If no, how many______  why? __________________________ |  | Yes/No | Yes/No | Yes/No  If no, how many______  Why? ______________________ |  |
| **Date** | Yes/No | Yes/No  If no, how many______  why? __________________________ |  | Yes/No | Yes/No | Yes/No  If no, how many______  Why? ______________________ |  |
| **Date** | Yes/No | Yes/No  If no, how many______  why? __________________________ |  | Yes/No | Yes/No | Yes/No  If no, how many______  Why? ______________________ |  |
| **Date** | Yes/No | Yes/No  If no, how many______  why? __________________________ |  | Yes/No | Yes/No | Yes/No  If no, how many______  Why? ______________________ |  |
| **Date** | Yes/No | Yes/No  If no, how many______  why? __________________________ |  | Yes/No | Yes/No | Yes/No  If no, how many______  Why? ______________________ |  |
| **Date** | Yes/No | Yes/No  If no, how many______  why? __________________________ |  | Yes/No | Yes/No | Yes/No  If no, how many______  Why? ______________________ |  |
| **Date** | Yes/No | Yes/No  If no, how many______  why? __________________________ |  | Yes/No | Yes/No | Yes/No  If no, how many______  Why? ______________________ |  |
| **Date** | Yes/No | Yes/No  If no, how many______  why? __________________________ |  | Yes/No | Yes/No | Yes/No  If no, how many______  Why? ______________________ |  |
| **Date** | Yes/No | Yes/No  If no, how many______  why? __________________________ |  | Yes/No | Yes/No | Yes/No  If no, how many______  Why? ______________________ |  |
| **Date** | Yes/No | Yes/No  If no, how many______  why? __________________________ |  | Yes/No | Yes/No | Yes/No  If no, how many______  Why? ______________________ |  |
| **Date** | Yes/No | Yes/No  If no, how many______  why? __________________________ |  | Yes/No | Yes/No | Yes/No  If no, how many______  Why? ______________________ |  |
| **Date** | Yes/No | Yes/No  If no, how many______  why? __________________________ |  | Yes/No | Yes/No | Yes/No  If no, how many______  Why? ______________________ |  |
| **Date** | Yes/No | Yes/No  If no, how many______  why? __________________________ |  | Yes/No | Yes/No | Yes/No  If no, how many______  Why? ______________________ |  |
| **Date** | Yes/No | Yes/No  If no, how many______  why? __________________________ |  | Yes/No | Yes/No | Yes/No  If no, how many______  Why? ______________________ |  |

**Supplementary file three. Unadjusted analysis for primary and secondary outcome measures mean (standard deviation) and interactions**

|  |  | **Radial extracorporeal shockwave therapy** |  | **Sham** | **Between group unadjusted difference**  **Coefficient (95% confidence interval)** | **P value** |
| --- | --- | --- | --- | --- | --- | --- |
| **Outcome** | **Week** | **Unadjusted mean (standard deviation)** |  | **Unadjusted mean (standard deviation)** |  |  |
| **Victorian institute of sports assessment - Achilles questionnaire** | 0 | 46.3 (16) |  | 47.7 (16) |  |  |
|  | 6 | 61.7 (16.2) |  | 58.8 (17.1) | 3 (-4.6 – 10.5) | (P=0.44) |
|  | 12 | 67.8 (15.6) |  | 63.3 (15.6) | 4.6 (-2.5 – 11.6) | (P=0.20) |
| **Visual analogue scale** | 0 | 3.1 (2.1) |  | 3.7 (2.1) |  |  |
|  | 6 | 2.7 (2.1) |  | 3.2 (2.3) | -0.5 (-1.5 – 0.5) | (P=0.33) |
|  | 12 | 2.7 (2.3) |  | 3 (2.4) | -0.3 (-1.4 -0.8) | (P=0.58) |
| **7-day Recall Physical Activity Questionnaire** | 0 | 203.9 (60.9) |  | 209 (60.9) |  |  |
|  | 6 | 216.4 (61.6) |  | 185.5 (64.6) | 31.5 (2.8 – 60.1) | (P=0.03) |
|  | 12 | 214.3 (55.4) |  | 214.3 (56) | 0.05 (-25.3 – 25.4) | (P=0.99) |
| **7-day Recall Physical Activity Questionnaire-mets** | 0 | 246.1 (125.7) |  | 283.3 (125.7) |  |  |
|  | 6 | 258.2 (127.3) |  | 229.3 (134.7) | 29.4 (-30 – 88.9) | (P=0.33) |
|  | 12 | 259.8 (119.3) |  | 251.9 (121) | 7.9 (-46.6 – 62.4) | (P=0.78) |
| **Tampa Scale of Kinesiophobia** | 0 | 25.5 (16.3) |  | 28.6 (16.3) |  |  |
|  | 6 | 23.4 (16.3) |  | 24.8 (17.2) | -1.4 (-9 – 6.3) | (P=0.73) |
|  | 12 | 24.1 (16.2) |  | 23.2 (16) | 0.9 (-6.4 – 8.2) | (P=0.81) |
| **Pain Catastrophising Scale** | 0 | 14.5 (10.5) |  | 15.3 (10.5) |  |  |
|  | 6 | 9.4 (10.5) |  | 7.3 (10.9) | 2.3 (-2.6 – 7.2) | (P=0.35) |
|  | 12 | 8 (10.3) |  | 8 (10.3) | 0.1 (-4.6 – 4.7) | (P=0.98) |
| **Pain Self-Efficacy Questionnnaire** | 0 | 46.8 (9.8) |  | 46.1 (9.8) |  |  |
|  | 6 | 51.7 (9.9) |  | 53.6 (10.4) | -2.2 (-6.8 – 2.4) | (P=0.35) |
|  | 12 | 52.9 (10.6) |  | 50.8 (10.6) | 2.1 (-2.7 – 6.9) | (P=0.38) |
| **EuroQol-5D-5L** | 0 | 0.81 (0.1) |  | 0.78 (0.1) |  |  |
|  | 6 | 0.88 (0.1) |  | 0.88 (0.1) | 0.01 (-0.06 – 0.08) | (P=0.81) |
|  | 12 | 0.91 (0.1) |  | 0.89 (0.1) | 0.02 (-0.04 – 0.08) | (P=0.52) |
| **EuroQol-5D-5L visual analogue scale** | 0 | 72.1 (17.5) |  | 71.1 (17.5) |  |  |
|  | 6 | 78.2 (17.6) |  | 81.5 (18.3) | -3.6 (-11.8 – 4.6) | (P=0.38) |
|  | 12 | 81.9 (18.1) |  | 76.4 (18) | 5.6 (-2.6 – 13.8) | (P=0.18) |
| **Productivity Cost Questionnaire_presenteesim** | 0 | - |  | - |  |  |
|  | 6 | 3093.3 (13668.6) |  | 0 |  |  |
|  | 12 | 2141.7 (11843.6) |  | 2379.5 (11843.6) | -237.8 (-5607.1 – 5131.5) | (P=0.09) |
| **Productivity Cost Questionnaire _unpaid** | 0 | - |  | - |  |  |
|  | 6 | 383.1 (1372.3) |  | 28.1 (173.1) |  |  |
|  | 12 | 413.8 (904.4) |  | 6.6 (904.4) | 407.2 (-2.8 – 817.2) | (P=0.05) |

**Supplementary file four. Non serious adverse events explanations**

| **Radial extracorporeal shockwave therapy** | | **Sham** | |
| --- | --- | --- | --- |
| **6 week** | **12 week** | **6 week** | **12 week** |
| Shockwave treatment irritated my heel for 24hrs  Calf soreness from heel lifts at the start of doing this exercise. | My wife's immigration problems | the achilies pain became too much on 2 particular days to the point it affected my emotion coming to the point of feeling like crying | Pain after walking for too long |
| Personal events | Nothing | When hopping the pain and inflamation in my achilles and heel increase | the pain did not settle |
| Doing the weight bearing exercise | Increase in calf and foot pain | Increase in areas that experience pain | Got leg cramps, toes cramps |
| Pain in achilles | Very sore pain | Pain worsened at times undertaking the calf raise exercises | I had increased walking activity due to a planned event that was unexpectedly busy & so I was walking & on my feet for a lot longer than expected |
| Pain when I start the exercise so takes a little while to get going but once I get going it improves | I over exercised one day - walked too far which resulted in increased pain for the net 2 days, but settled down after that. | Achilles pain in the mornings | Heightened pain when initially performing leg raise exercises with weights of 25 kg. This did not improve over a couple of weeks so I reduced the load to 20 kg. Achilles is a little more sore the following morning but then settles down during the day. |
| Had pain in leg mussels | Pain in calf muscle due to muscle imbalance | I travelled on the spirit of Tasmania & this was uncomfortable & I had significant back & leg pain | Slight increase in pain due to playing 10 consecutive days of golf. |
| Unfortunately over a week ago they did something to my back. Think it is a sprained Richmond v and I have been in terrible pain and unable to do much | Extra pain due to over exercising and the extra demands at work | An increase in muscle tightness due to weighted calf raises. |  |
| Struggle with certain weight bearing exercises | some achilles pain after running | Sharp pain when increasing weight on prescribed exercises |  |
| Basically if I have worked from home I have been relatively pain free, if I have worked in the office I have ended up with considerable pain by the end of the day, and this prevents me from doing my exercises the next day.  I couldn't avoid being in the office the past 6 weeks because of a project go live however I am dedicated to being at home now for the next few weeks | Other Achilles’ tendon has been playing up and making recovery challenging | Pain in achilles due to overloading |  |
|  | I skipped exercise as traveling/busy and had more pain |  |  |
|  | While walking but after about 10 minutes it eases so while it warm it’s not so bad but when I’m home and it cools down the pain is back. It also wakes me when asleep. |  |  |
|  | I has to have unexpected surgery unrelated to my Achilles injury. |  |  |
|  | About 5 weeks I started to feel pain in the ball of my right foot. It progressed to quite a bit of swelling and increasing pain. Even though I did ice, compression, elevation & anti-inflamms the pain seemed to get worse as I continued the rehab work.   I reduced the load and intensity of the rehab exercises as well as the time spent walking but the problem persisted. Over the last few weeks I have been unable to bear my body weight on my right foot which has prevented me from doing any of the rehab exercises.  This has resulted in increased stiffness in the tendon as I am only able to perform very minor activity.  As the pain was not reducing even with rest I got ultrasound and xrays of the right foot. The diagnosis is acute bursitis of the 2nd and 3rd metatarsophalangeal joints on my right foot |  |  |
|  | Pain increased in my left leg and I had to stop the running as it was increasing and required a greater period of time to get up and warm up my tendon in the morning |  |  |
